# Stillbirth Trends and Determinants: A Six-Year Registry-Based Analysis From Two District Hospitals in the United Kingdom (2019-2024)

**DOI:** 10.7759/cureus.94706

**Published:** 2025-10-16

**Authors:** Rupa Brahma, Manjula Samyraju, Lesley Carline

**Affiliations:** 1 Obstetrics and Gynecology, Peterborough City Hospital, Peterborough, GBR

**Keywords:** ethnicity, maternal obesity, neonatal deaths, reduced fetal movement, retrospective cohort, stillbirth

## Abstract

Introduction: Despite advances in obstetric care, stillbirth remains a major public health concern. Understanding local patterns and risk factors is essential for developing targeted strategies for prevention. This study aimed to review the demographic and clinical characteristics of stillbirths across two district hospitals in the United Kingdom over a six-year period.

Methods: A retrospective secondary analysis was conducted using registry-based surveillance data of all stillbirths occurring between 2019 and 2024 at two hospitals under the same healthcare trust. Data on maternal demographics, gestational age at stillbirth, and associated risk factors were extracted and analyzed. Descriptive statistics were used to identify key trends. As this was a secondary analysis of de-identified data, ethical approval was waived.

Results: A total of 118 stillbirths were documented during the study period. The majority occurred in women aged >30 years (n=68, 57.6%), with the highest proportion in those aged >35 years (n=39, 33.0%). A significant number of stillbirths occurred at >36 weeks’ gestation (n=42, 35.6%). White women accounted for the majority of cases (n=84, 71.2%), although notable trends were also observed among Asian and Black ethnicities in specific years. Reduced fetal movements (n=50, 42.4%) and high maternal BMI (n=31, 26.3%) were the most frequently reported modifiable risk factors.

Conclusion: This study highlights ongoing challenges in stillbirth prevention and emphasizes the importance of timely detection of fetal compromise and targeted interventions for at-risk groups. Regular institutional reviews are essential for guiding quality improvement efforts.

## Introduction

Stillbirth, defined as the birth of a baby showing no signs of life at or after 24 weeks of gestation, remains one of the most devastating outcomes in obstetrics, representing a significant global health challenge with profound implications for families, healthcare providers, and health systems [1,2]. According to the report of Mothers and Babies: Reducing Risk through Audits and Confidential Enquiries across the UK (MBRRACE-UK), the stillbirth rate was approximately 3.22 per 1,000 births nationally in the United Kingdom in 2023, which was slightly lower than 3.35 per 1,000 births in 2022 and 3.54 per 1,000 births in 2021 [3]. Despite substantial advances in antenatal care and fetal monitoring technologies, stillbirth rates have demonstrated only modest improvement over recent decades, highlighting the urgent need for comprehensive analysis and targeted interventions.

Understanding the underlying risk factors and causes of stillbirth is vital to developing targeted interventions and improving maternal-fetal outcomes. The multifactorial aetiology of stillbirth encompasses a complex interplay of maternal, fetal, and placental factors [4]. Major risk factors include advanced maternal age, pre-existing medical conditions such as diabetes mellitus and hypertension, pregnancy complications including pre-eclampsia and fetal growth restriction, and social determinants including socioeconomic deprivation and ethnic disparities [5-7]. Placental dysfunction, congenital anomalies, and maternal infections also contribute significantly to perinatal mortality [8,9]. However, in a substantial proportion of cases, the exact cause remains unexplained even after thorough investigation, including postmortem examination and placental histology.

Reviews at the institutional level offer valuable insights into local patterns of stillbirth, identify areas for improvement, and contribute to evidence-based practice development. By systematically reviewing stillbirth cases, hospitals can identify preventable factors, evaluate adherence to clinical guidelines, and inform training and resource allocation. This would also facilitate comparison with national data and establish follow-up measures in compliance with Saving Babies’ Lives for reducing stillbirth. This study presents a retrospective review of stillbirths over a six-year period at two district hospitals (under the same trust) in the United Kingdom, which aimed to characterize the demographic and clinical profile of affected mothers, assess the distribution of gestational age and birth weight at the time of stillbirth, and identify prevalent maternal and fetal risk factors. Through this analysis, we seek to contribute to the ongoing efforts to reduce stillbirth rates and enhance perinatal outcomes and contribute to the broader understanding of stillbirth epidemiology in contemporary obstetric care.

## Materials and methods

Study design and setting

This retrospective secondary data analysis was conducted using six years of registry-based surveillance data (2019-2024) from two district hospitals (denoted as Hospital A and Hospital B) under the same healthcare trust in the United Kingdom. The study has been reported in accordance with the Strengthening the Reporting of Observational Studies in Epidemiology (STROBE) guidelines.

Data source

Data were obtained from institutional birth records routinely maintained by the hospitals as part of their perinatal surveillance efforts. Additional data sources included the Perinatal Mortality Review Tool (developed by a collaboration led by MBRRACE-UK) and incident reports.

Study population and eligibility criteria

All recorded cases of stillbirth, defined as the delivery of a baby with no signs of life at or after 24 weeks of gestation, were included during the study period. Cases with incomplete demographic or clinical information were excluded from the analysis.

Study parameters

The dataset included maternal demographic information (age, ethnicity), gestational age at the time of stillbirth, and clinical details such as the presence of known risk factors (e.g., diabetes, hypertension, reduced fetal movements, high BMI, congenital infections, smoking, twin pregnancy, previous stillbirth, and small-for-gestational-age fetus). Year-wise distribution of stillbirths, as well as stratification by gestational age and maternal ethnicity, was also extracted.

Ethical considerations

As the study was based on anonymized secondary data routinely collected for audit and surveillance purposes and no patient identifiers were used, ethical approval was waived in accordance with institutional policy.

Statistical analysis

The dataset was cleaned and compiled in Microsoft Excel 2016 and analyzed using IBM SPSS Statistics for Windows, Version 25.0 (Released 2017; IBM Corp., Armonk, NY, USA). Descriptive statistics were used to summarize the data. Categorical variables were expressed as absolute frequencies and percentages. Year-wise trends were depicted using line and stacked bar diagrams. No inferential statistical testing was performed, as the primary aim was descriptive exploration of patterns and risk factor profiles.

## Results

**Figure 1:**
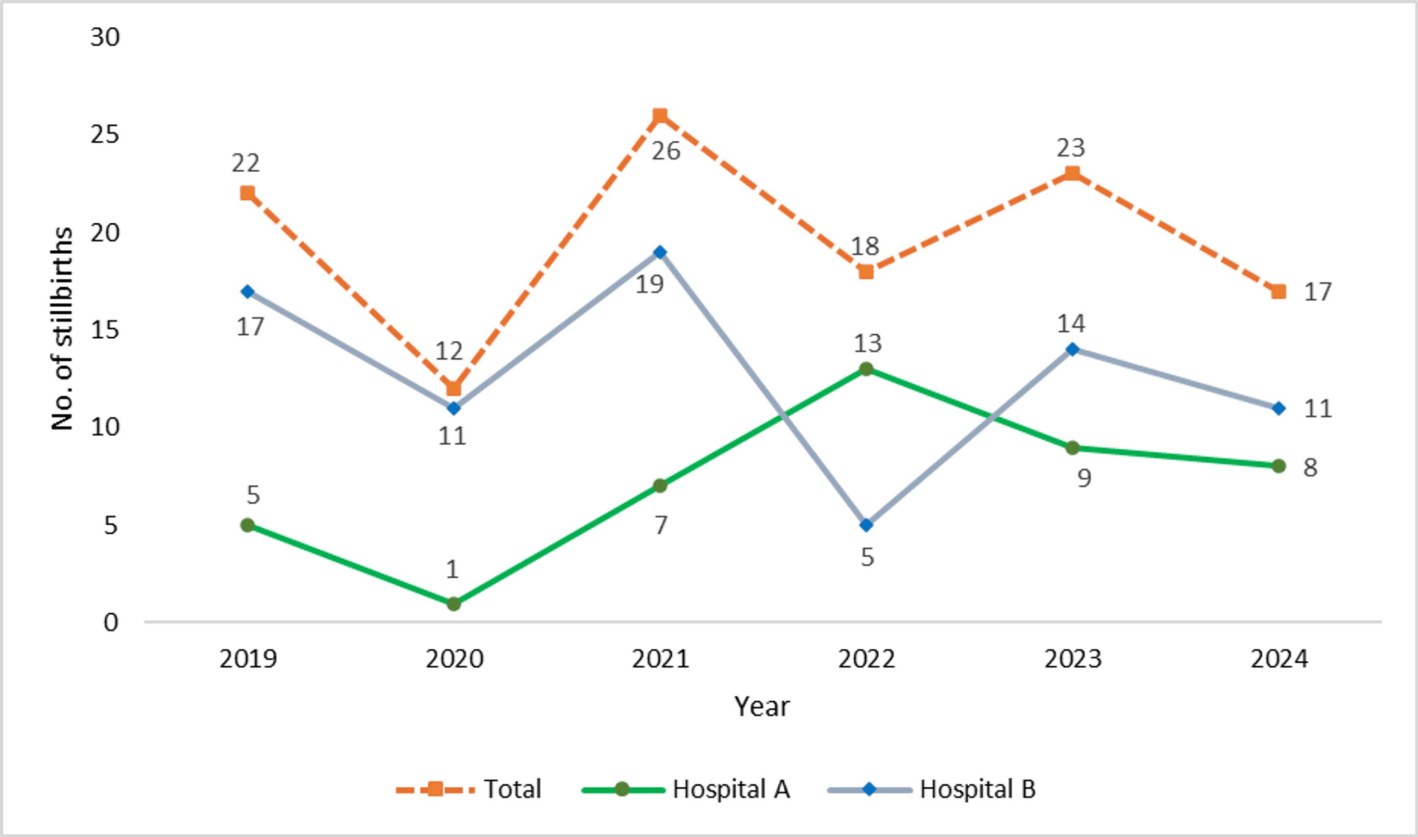
Line diagram showing number of stillbirths by year in both the study hospitals (together and separately).

Over the six-year study period (2019-2024), a total of 118 stillbirths were reported across the two district hospitals. The annual trend revealed notable fluctuations in stillbirth numbers, with the highest count observed in 2021 (n=26) and the lowest in 2020 (n=12). A sharp decline was seen from 2019 (n=22) to 2020, followed by a peak in 2021 and variable figures in subsequent years: 18 cases in 2022, 23 in 2023, and 17 in 2024. Distribution between hospitals showed that Hospital B consistently reported more cases than Hospital A, except in 2022 when Hospital A had a higher count (Figure 1).

The age distribution indicated that the majority of stillbirths occurred among women aged above 30 years. Specifically, 33.0% (n=39) were >35 years, and 24.6% (n=29) were between 31 and 35 years. Women aged 26-30 years constituted 27.1% (n=32), and those aged <25 years made up 15.3% (n=18) of the cases (Table 1).

**Table 1:** Baseline demographic and obstetric characteristics of stillbirths (N=118).

Characteristics	Categories	Number	Frequency
Age	<25 years	18	15.3
	26-30 years	32	27.1
	31-35 years	29	24.6
	>35 years	39	33.0
Ethnicity	Asian	20	16.9
	Black	14	11.9
	White	84	71.2
Gestational age at birth	>24 to ≤27 weeks	26	22.0
	>27 to ≤32 weeks	28	23.7
	>32 to ≤36 weeks	22	18.6
	>36 weeks	42	35.6

**Figure 2:**
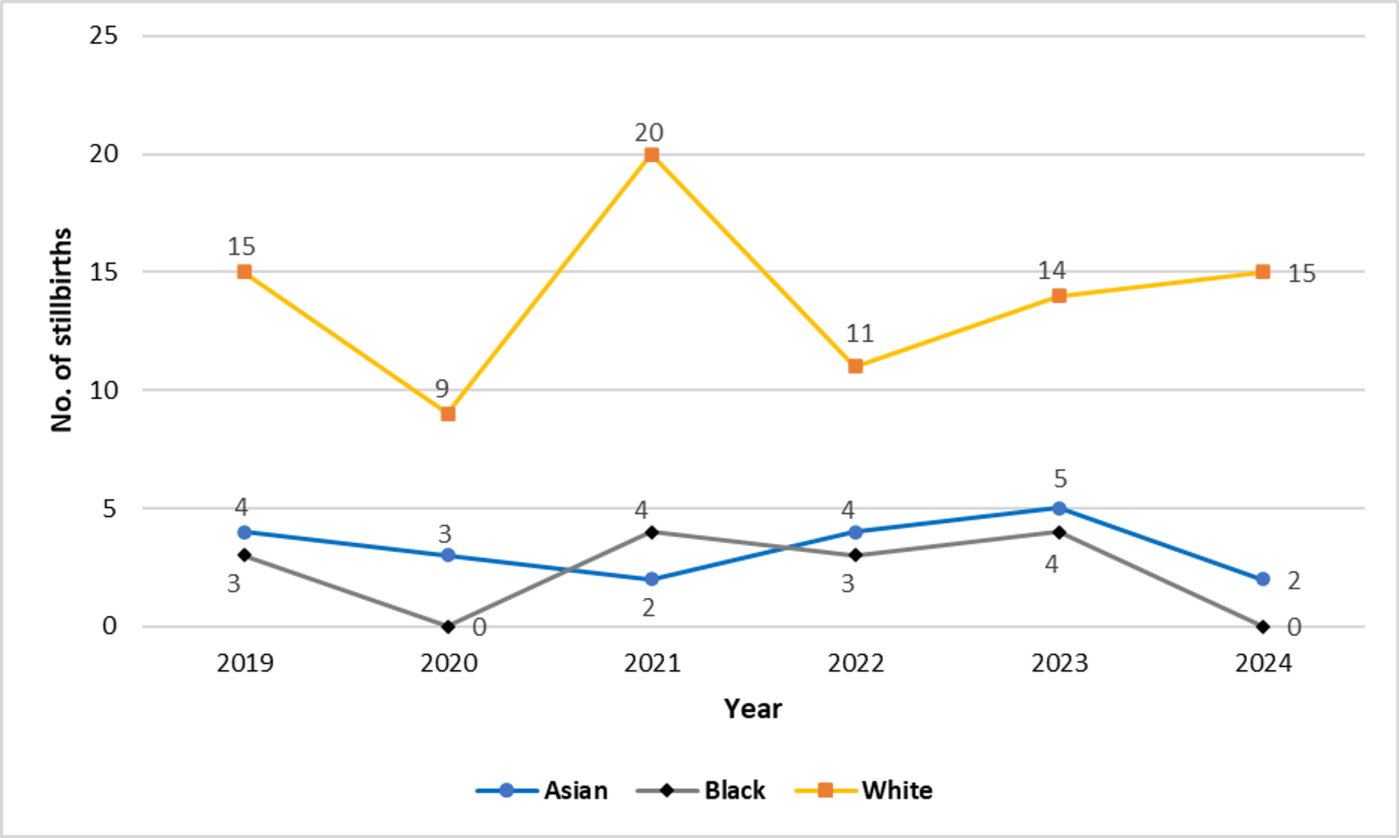
Line diagram showing number of stillbirths by year and ethnicity.

Ethnic distribution showed that White women accounted for the largest proportion of stillbirths (71.2%, n=84), followed by Asian (16.9%, n=20) and Black women (11.9%, n=14). Year-wise analysis (Figure 2) indicated that stillbirths among White women remained consistently high across all years, peaking in 2021 (n=20) and again in 2024 (n=15). In contrast, stillbirths among Asian and Black women remained relatively lower and stable, with occasional peaks (e.g., four Black stillbirths in both 2021 and 2023; five Asian stillbirths in 2023).

**Figure 3:**
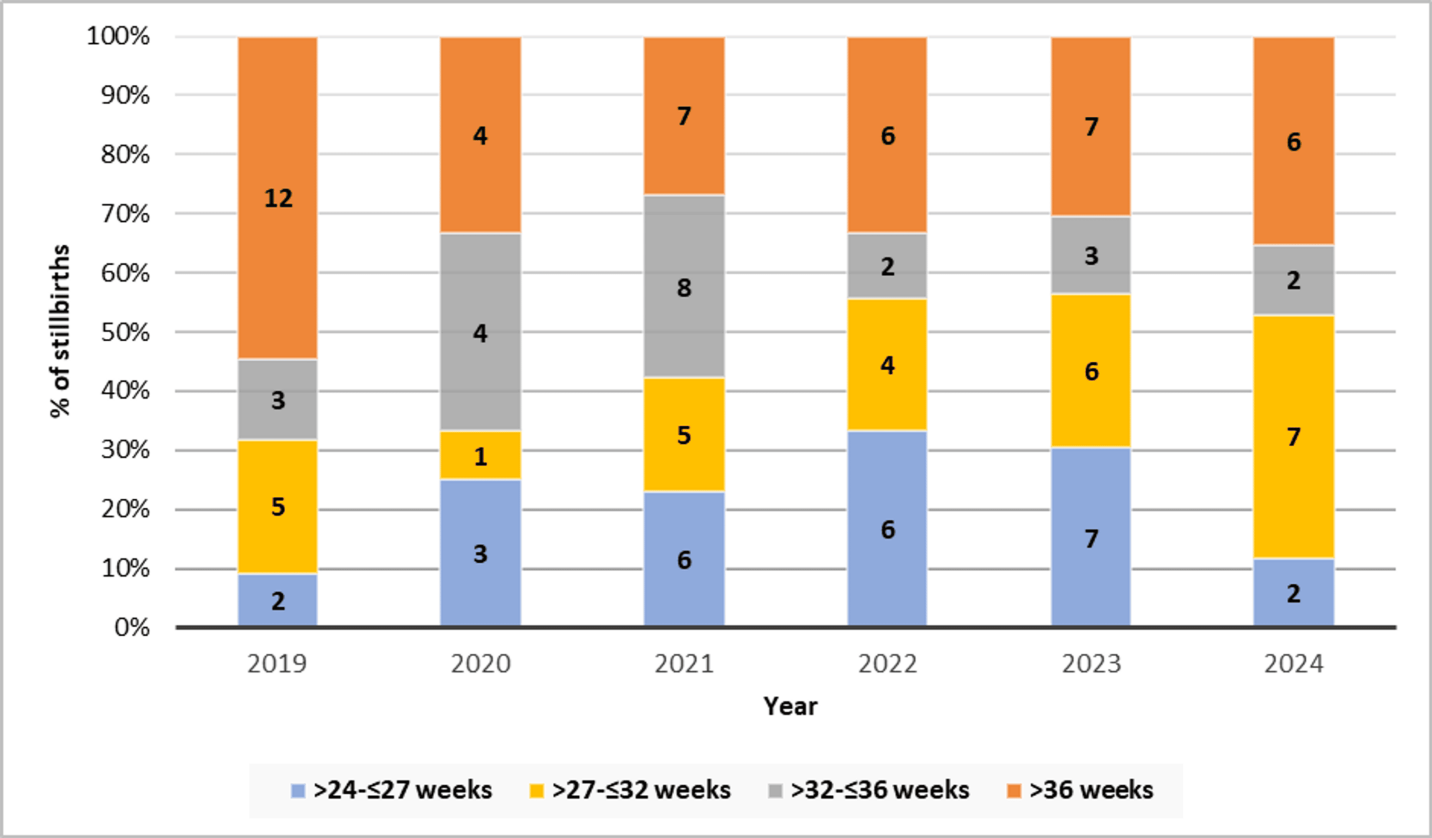
Stacked bar diagram showing number of stillbirths by year and gestational age

Gestational age analysis showed that 35.6% (n=42) of stillbirths occurred after 36 weeks of gestation, followed by 23.7% (n=28) at >27 to ≤32 weeks, 22.0% (n=26) at >24 to ≤27 weeks, and 18.6% (n=22) at >32 to ≤36 weeks (Table 1). Figure 3 presents the distribution of stillbirths based on gestational age and year. In 2019, most stillbirths occurred beyond 36 weeks (n=12), while in 2021, a broader spread was noted across all gestational categories. In 2024, the majority were clustered in the >27 to 32 weeks range (n=7), whereas >36 weeks continued to show persistent contributions to the annual burden across all years.

The analysis of risk factors (Table 2) revealed that reduced fetal movements were the most commonly documented concern prior to stillbirth, present in 42.4% (n=50) of cases. High maternal BMI (>30 kg/m²) was the next most prevalent factor, observed in 26.3% (n=31). Diabetes, hypertension or pre-eclampsia, and congenital infections each accounted for 11.0% (n=13). Other less frequent but relevant factors included small-for-gestational age (6.8%, n=8), twin pregnancy (4.2%, n=5), smoking (4.2%, n=5), and a previous history of stillbirth (2.5%, n=3).

**Table 2:** Occurrence of established risk factors of stillbirths (N=118).

Risk factors	Frequency	Percentage
Diabetes	13	11.0
Previous history stillbirth	3	2.5
Twin pregnancy	5	4.2
High body mass index (>30 kg/m^2^)	31	26.3
Smoking	5	4.2
Hypertension/ preeclampsia	13	11.0
Small-for-gestational age	8	6.8
Congenital infections	13	11.0
Reduced fetal movements	50	42.4

## Discussion

This six-year retrospective analysis of stillbirths across two district hospitals in the United Kingdom offers critical insights into the demographic, clinical, and temporal patterns of stillbirths. The study findings echo several national trends while also highlighting local challenges and opportunities for intervention. The total number of stillbirths varied annually, peaking in 2021 (n=26) and reaching a low in 2020 (n=12). This variation may partially reflect the indirect impacts of the coronavirus disease 2019 (COVID-19) pandemic on antenatal care access, maternal stress, and health system priorities, factors previously associated with adverse pregnancy outcomes [10]. Interestingly, while MBRRACE-UK reported a national decline in stillbirth rates in 2023 to 3.22 per 1,000 births [3], our institution-level data suggest that local variability persists, underscoring the need for trust-level quality improvement audits.

In terms of maternal age, the majority of stillbirths were observed in women over the age of 30, with 33.0% occurring in women over 35 years. This finding aligns with previous studies that have consistently linked advanced maternal age with increased stillbirth risk, often attributed to a higher prevalence of comorbidities, placental insufficiency, and chromosomal abnormalities [5,11]. Notably, the adjusted mortality rates provided in the MBRRACE-UK 2023 report account for maternal age as a key confounder, reinforcing its importance as a demographic determinant [3].

Ethnic disparities were evident, with White women comprising 71.2% of stillbirths, followed by Asian (16.9%) and Black (11.9%) women. On the contrary, the MBRRACE-UK 2023 national report confirmed that babies of Black ethnicity remain more than twice as likely to be stillborn than White babies (5.84 vs 2.71 per 1,000 births), and stillbirth rates among Asian babies increased by nearly 10% from the previous year [3]. These persistent disparities are believed to stem from a complex interplay of structural racism, unequal access to healthcare, cultural barriers, and higher prevalence of underlying risk factors in these communities [12-14].

Gestational age analysis in our study showed that 35.6% of stillbirths occurred after 36 weeks, while 46% occurred between 24 and 32 weeks. These figures align with the MBRRACE-UK national data, indicating that 76% of stillbirths in 2023 occurred before 37 weeks of gestation [3]. The considerable burden of late preterm and term stillbirths underlines missed opportunities for timely intervention and monitoring. The spike in post-36-week stillbirths suggests a pressing need to optimize fetal surveillance protocols and decision-making around induction of labor for high-risk pregnancies.

The most prevalent modifiable clinical risk factor identified was reduced fetal movements, reported in 42.4% of cases. This finding reinforces the established association between maternal perception of reduced fetal activity and fetal compromise [15-17]. The national “Saving Babies' Lives” care bundle emphasizes timely response to reduced fetal movement as a critical component in reducing stillbirths [18]. High maternal BMI (>30 kg/m²) was the second most common factor (26.3%), a pattern consistent with evidence linking obesity with placental dysfunction, gestational diabetes, and hypertensive disorders, all of which elevate stillbirth risk [19].

Hypertensive disorders, diabetes, and congenital infections were each present in 11.0% of stillbirths in our cohort, aligning with the multifactorial pathophysiology of stillbirth highlighted in previous literature [5,6,19]. Although congenital anomalies were not the primary focus of our study, their notable contribution (11.0%) underscores the importance of early anomaly screening and preconception counselling. Nationally, MBRRACE-UK reported that 8.4% of stillbirths were attributed to congenital anomalies, although many deaths remain unexplained due to incomplete postmortem or placental examination [3].

Despite the use of rigorous registry data, the absence of postmortem findings in our dataset limits the exploration of direct causes of death. MBRRACE-UK emphasized that in 2023, over one-third of stillbirths still had an unknown cause [3]. This calls for greater parental engagement in consenting to postmortem and enhanced support from bereavement teams.

A key strength of this study is its use of six years of registry-based data from two district hospitals, allowing for detailed exploration of temporal patterns, demographic profiles, and clinical risk factors associated with stillbirths. Additionally, the large dataset and inclusion of multiple years enhance the reliability of observed trends and provide valuable insights into local practice and disparities. However, there are notable limitations. As a retrospective secondary data analysis, the study relied on pre-recorded information, which may be subject to documentation biases and missing data. Importantly, we were unable to retrieve the total number of births at the two hospitals during the study period, which precluded the calculation of stillbirth rates. This limits our ability to make direct comparisons with national or regional stillbirth rates reported across the UK. Furthermore, the lack of detailed information on the causes of death, including postmortem and placental histology findings, restricted a deeper etiological classification of the stillbirths.

## Conclusions

Based on this six-year retrospective review of stillbirths at two district hospitals in the United Kingdom, we conclude that most stillbirths occurred in women over 30 years of age, at later gestational ages, and predominantly among White women. High maternal BMI and reduced fetal movements emerged as the most frequently observed modifiable risk factors. These findings underscore the urgent need to enhance maternal awareness and healthcare provider vigilance regarding the significance of RFM, even at the first presentation. Strengthening education for both patients and healthcare workers on recognizing and responding to RFM can play a critical role in timely intervention and prevention. The persistent burden of stillbirth in the UK calls for improved antenatal surveillance, targeted risk stratification, and proactive management strategies tailored to vulnerable populations. Finally, regular institutional audits and systematic data reviews remain essential for guiding evidence-based interventions and improving perinatal outcomes at the regional level.
